# Estrogen receptor coregulator binding modulator (ERX-11) enhances the activity of CDK4/6 inhibitors against estrogen receptor-positive breast cancers

**DOI:** 10.1186/s13058-019-1227-8

**Published:** 2019-12-26

**Authors:** Suryavathi Viswanadhapalli, Shihong Ma, Gangadhara Reddy Sareddy, Tae-Kyung Lee, Mengxing Li, Collin Gilbreath, Xihui Liu, Yiliao Luo, Uday P. Pratap, Mei Zhou, Eliot B. Blatt, Kara Kassees, Carlos Arteaga, Prasanna Alluri, Manjeet Rao, Susan T. Weintraub, Rajeshwar Rao Tekmal, Jung-Mo Ahn, Ganesh V. Raj, Ratna K. Vadlamudi

**Affiliations:** 1 Department of Obstetrics and Gynecology, University of Texas Health, San Antonio, TX 78229 USA; 2 Departments of Urology and Pharmacology, University of Texas Southwestern Medical Center at Dallas, Dallas, TX 75390 USA; 3 CDP Program, University of Texas Health Cancer Center, San Antonio, TX 78229 USA; 4 Department of Chemistry and Biochemistry, University of Texas at Dallas, Richardson, TX 75080 USA; 5 Simmons Cancer Center, University of Texas Southwestern Medical Center at Dallas, Dallas, TX 75390 USA; 6 Department of Radiation Oncology, University of Texas Southwestern Medical Center at Dallas, Dallas, TX 75390 USA; 7 Department of Cell Systems and Anatomy, University of Texas Health, San Antonio, TX 78229 USA; 8 Department of Biochemistry and Structural Biology, University of Texas Health, San Antonio, TX 78229 USA

**Keywords:** Estrogen receptor (ER), ER coregulators, Breast cancer, ER coregulator modulator, Therapy-resistant breast cancer, CDK4/6 inhibitor, Palbociclib

## Abstract

**Background:**

CDK4/6 inhibitors in combination with endocrine therapy (AE/AI/SERDs) are approved for the treatment of ER+ advanced breast cancer (BCa). However, not all patients benefit from CDK4/6 inhibitors therapy. We previously reported a novel therapeutic agent, ERX-11, that binds to the estrogen receptor (ER) and modulates ER-coregulator interactions. Here, we tested if the combination of ERX-11 with agents approved for ER+ BCa would be more potent.

**Methods:**

We tested the effect of combination therapy using BCa cell line models, including those that have acquired resistance to tamoxifen, letrozole, or CDK4/6 inhibitors or have been engineered to express mutant forms of the ER. In vitro activity was tested using Cell Titer-Glo, MTT, and apoptosis assays. Mechanistic studies were conducted using western blot, reporter gene assays, RT-qPCR, and mass spectrometry approaches. Xenograft, patient-derived explants (PDEs), and xenograft-derived explants (XDE) were used for preclinical evaluation and toxicity.

**Results:**

ERX-11 inhibited the proliferation of therapy-resistant BCa cells in a dose-dependent manner, including ribociclib resistance. The combination of ERX-11 and CDK4/6 inhibitor was synergistic in decreasing the proliferation of both endocrine therapy-sensitive and endocrine therapy-resistant BCa cells, in vitro, in xenograft models in vivo, xenograft-derived explants ex vivo, and in primary patient-derived explants ex vivo. Importantly, the combination caused xenograft tumor regression in vivo. Unbiased global mass spectrometry studies demonstrated profound decreases in proliferation markers with combination therapy and indicated global proteomic changes in E2F1, ER, and ER coregulators. Mechanistically, the combination of ERX-11 and CDK4/6 inhibitor decreased the interaction between ER and its coregulators, as evidenced by immunoprecipitation followed by mass spectrometry studies. Biochemical studies confirmed that the combination therapy significantly altered the expression of proteins involved in E2F1 and ER signaling, and this is primarily driven by a transcriptional shift, as noted in gene expression studies.

**Conclusions:**

Our results suggest that ERX-11 inhibited the proliferation of BCa cells resistant to both endocrine therapy and CDK4/6 inhibitors in a dose-dependent manner and that the combination of ERX-11 with a CDK4/6 inhibitor may represent a viable therapeutic approach.

## Introduction

Breast cancer (BCa) is the most common cancer in women. The majority of BCa (70%) is estrogen receptor-positive (ER+). ER is activated by estrogenic ligands like estradiol (E2). ER signaling plays a key role in BCa cell cycle progression from G_1_ to S phase and is a critical molecular driver of BCa tumorigenesis. The primary therapeutic options for patients with systemic ER+ BCa are drugs targeting ER signaling, using either competitive antagonists like antiestrogens (AE) or antiestrogens like aromatase inhibitors (AI). However, most patients develop resistance to these endocrine drugs, and disease recurrence and progression is common [1, 2]. Importantly, the majority of endocrine therapy-resistant tumors retain ER signaling, through either mutation in the ER, alternative ligands, or altered coregulator profiles.

ER signaling requires ER interaction with oncogenic coregulator proteins [3, 4]. Over one third (38%) of ER coregulators are over-expressed in BCa [4–6] including oncogenic coregulators SRC3 [7, 8], SRC2 [9], and PELP1 [10]. These deregulated coregulators contribute to mammary tumorigenesis [6], therapy resistance, and metastases [11–14]. Alterations in the concentration or activity of oncogenic coregulators enable ER signaling from AE-ER complexes, effectively converting the antagonist to an agonist [15, 16]. Recent studies also showed ER mutations lead to constitutive activity by enhancing ER-coregulator interactions with reduced sensitivity to ER antagonists, and mutations such as Y537S contribute to fulvestrant resistance in vivo [17].

Transcriptional and post-translational regulation of ER coregulators [18] may play a critical role in ER regulation of cell cycle progression. The activity of cyclin-dependent kinases (CDKs) [19] is known to regulate several coregulators via phosphorylation. Luminal breast cancer commonly exhibits cyclin D1 and CDK4 amplification [20], while endocrine therapy-resistant tumors often exhibit deregulation of the CDK4/6 pathway [21]. Recently, the combination of CDK4/6 inhibitors and endocrine therapy (AE/AI/SERDs) has been approved for the treatment of ER+ advanced BCa [22]. Three selective CDK4/6 inhibitors (palbociclib, ribociclib, and abemaciclib) bind to the ATP-binding pocket of CDK4/6 and produce a cytostatic effect in combination with agents that target ER signaling [21]. However, some patients do not benefit from this combination, and the emergence of resistance in responders is common.

We recently reported the development of a small organic molecule, ERX-11 [23] (ER coregulator binding modulator-11), that uniquely interacts with ER and blocks the interaction of selective oncogenic coregulators with ER. ERX-11 was shown to block ER signaling and ER-driven proliferation in therapy-sensitive and therapy-resistant ER+ BCa. Since endocrine therapy-resistant tumors retain functional ER signaling via oncogenic coregulator proteins, we reasoned that the combination of ERX-11 with CDK4/6 inhibitors could be synergistic and prevent the emergence of resistant phenotypes.

In this manuscript, we tested and demonstrated the utility of the combination of ERX-11 and CDK4/6 inhibitors (palbociclib, abemaciclib, ribociclib) in treating therapy-sensitive and therapy-resistant advanced BCa. These studies provide the preclinical rationale for this combination therapy.

## Materials and methods

### Cell lines

Human BCa cells MCF-7, ZR-75, and T-47D were obtained from the American Type Culture Collection (ATCC, Manassas, VA). ZR-75-ESR1-MT-D538G and ZR-75-ESR1-MT-Y537S cells were described earlier and were cultured in RPMI media containing 10% fetal bovine serum (FBS) [23]. Ribociclib-resistant cells (MCF-7/RR) were described earlier [24]. MCF-7/TamR cells [25] and MCF-7/LTLTca cells [26] were described earlier. MCF-7/LTLTca and MCF-7/TamR cells were cultured in phenol red-free RPMI medium containing 5% dextran-coated charcoal-treated FBS (DCC-FBS) supplemented with either 1 μmol/L of letrozole or 1 μmol/L of tamoxifen, respectively. All the model cells utilized are free of mycoplasma contamination. Additionally, STR DNA profiling of the cells was used to confirm the identity using UTHSA and UT Southwestern core facilities.

### Reagents

Letrozole, 17-β-estradiol, (Z)-4-hydroxytamoxifen, and androstenedione were purchased from Sigma (St. Louis, MO). Palbociclib was purchased from Cayman Chemical (MI, USA). Ribociclib (HY-15777) and abemaciclib (HY-16297A) were purchased from MedChem Express LLC (Monmouth Junction, NJ). Ki-67 anti-human clone MIB-1 antibody (cat#M7240) was purchased from Dako (Carpinteria, CA). The PELP1, SRC3, and SRC1 antibodies were purchased from Bethyl Laboratories (Montgomery, TX). The GAPDH, p-ERK1/2, ERK1/2, p-4EBP1, 4EBP1, p-mTOR(S2448), mTOR, p-p70S6K(T389), p70S6K, p21, PARP, and p65 antibodies were purchased from Cell Signaling Technology (Beverly, MA). The E2F1 and FOXM1 antibodies were purchased from Santa Cruz Biotechnology. The anti-ER alpha and anti-phospho-histone H2A.X (Ser139) antibodies were purchased from Millipore. The cyclin D1, β-actin, and all secondary antibodies were purchased from Sigma. ERX-11 was synthesized by following the previously reported procedure [23].

### Cell viability and colony formation assays

The effects of ERX-11 and CDK4/6 inhibitors on cell viability were measured using the MTT cell viability assay in 96-well plates. BCa cells were seeded in 96-well plates (1 × 10^3^ cells/well) in phenol red-free RPMI medium containing 5% DCC-FBS. After an overnight incubation, cells were treated with varying concentrations of the ERX-11, palbociclib, abemaciclib, ribociclib, or combination of ERX-11 with each of CDK4/6 inhibitors in the presence or absence of estrogen (E2) (10^−8^ M) for 7 days. For some experiments, cell viability was also measured using Cell Titer-Glo luminescent cell viability assay (Promega) in 96-well, flat, clear-bottom, opaque-wall microplates according to the manufacturer’s protocol. For colony formation assays, MCF-7, ZR-75, ZR-75-ESR1-MT-Y537S, and ZR-75-ESR1-MT-D538G model cells (500 cells/well) were seeded in 6-well plates, treated with indicated drugs, and allowed to grow for 14 days. The cells were fixed in ice-cold methanol and stained with 0.5% crystal violet solution. Pixels above a threshold of 5000 in their intensity value were counted.

### Western blotting and immunoprecipitation

Western blotting and immunoprecipitation were performed as described previously [23]. Briefly, for western blot, whole-cell lysates were prepared using RIPA buffer containing protease and phosphatase inhibitors (Sigma Chemical Co.). Total proteins (50 μg) were mixed with SDS sample buffer and subjected to SDS-PAGE. Blots were developed using the ECL kit (Thermo Scientific, Waltham, MA).

### Cell cycle analysis

MCF-7 and MCF-7/TamR cells were seeded in 100-mm culture plates, and after overnight incubation, cells were treated with either vehicle, ERX-11 (1 μM), palbociclib (250 nM), or combination in the presence of estrogen (E2) (10^−8^ M) for 48 h. Cells were then trypsinized and harvested in PBS, followed by fixation in ice-cold 70% ethanol for 30 min at 4 °C. Cells were washed again with PBS and stained with a mixture of 50 μg/mL propidium iodide and 50 μg/mL RNase A. The PI-stained cells were subjected to flow cytometry using a FACS Calibur (BD Biosciences).

### Animal studies

All animal experiments were performed after obtaining UTHSA IACUC approval using methods in the approved protocol. For xenograft studies, 2 × 10^6^ MCF-7/TamR and MCF-7/LTLT model cells were mixed with an equal volume of Matrigel and implanted in the mammary fat pads of 6-week-old female nude mice implanted with tamoxifen and androstenedione pellets, respectively as described [27]. When the tumor was established, it was dissected into small pieces and they were again implanted subcutaneously into the nude mice. Once the tumors reached a measurable size, mice were randomly selected to receive vehicle and treatment with ERX-11 (10 mg/kg/day orally), palbociclib (50 mg/kg/day orally), or in combination (*n* = 4–7 tumors). Tumor growth was measured with a caliper at 3–4-day intervals. At the end of each experiment, the mice were euthanized, and the tumors were removed, weighed, and processed for immunohistochemical staining.

### Xenograft-derived explant and patient-derived explant studies

For xenograft-derived explant (XDE) studies, xenograft tumors of ZR-75-ESR1-MT-D538G, ZR-75-ESR1-MT-Y537S, and MCF-7/LTLT were initially established in SCID mice as described above, and earlier published patient-derived explant (PDE) protocol [23] was adapted for XDE assay. When the tumor reached 700 mm^3^, they were dissected into 2-mm cubes. Tumor samples were incubated on gelatin sponges for 24 h in culture medium containing 10% FBS, followed by treatment with either vehicle, 1 μM ERX-11, 500 nM palbociclib, or combination for 72 h. For PDE studies, excised tissue samples were processed and cultured ex vivo as previously described [23]. De-identified patient tumors were obtained from the UTSW Tissue Repository after institutional review board approval (STU-032011-187, 28). Briefly, tumor samples were incubated on gelatin sponges for 24 h in culture medium containing 10% FBS, followed by treatment with either vehicle, 10 μM ERX-11, 250 nM palbociclib, or combination for 72 h. Representative tissues were fixed in 10% formalin at 4 **°**C overnight and subsequently processed into paraffin blocks. The sections were then processed for immunohistochemical analysis.

### RT-qPCR

Reverse transcription (RT) reactions were performed by using SuperScript III First Strand kit (Invitrogen, Carlsbad), according to the manufacturer’s protocol. Real-time PCR was done using SYBR Green on an Illumina Real-Time PCR system. Primer sequences of the genes used were obtained from Harvard Primer Bank (https://pga.mgh.harvard.edu/primerbank).

### Mass spectrometry-based DIA analyses of whole-cell lysates

MCF-7/TamR cells were treated with E2 (10^−8^ M) plus vehicle or ERX-11 (1 μM), palbociclib (250 nM), or the combination of ERX-11 and palbociclib. After 72 h of the treatment, cells were pelleted and snap-frozen and then lysed 5% SDS/50 mM TEAC (DIA). Protein concentrations were determined by EZQ Protein Quantitation kit (Thermo Fisher) and peptides by Pierce Quantitative Fluorometric Peptide Assay (Thermo Fisher). For DIA analyses, S-Traps (Protifi) were used for reduction/alkylation, tryptic digestion, and cleanup, starting with 100 μg of protein (yield 66.5 ± 7.5 μg). A pool was made of the 12 samples (3 replicates from each group), and 2 μg peptide aliquots were analyzed on the Lumos using gas-phase fractionation and 4-*m*/*z* windows (120k resolution for precursor scans, 15k for product ion scans, all in the orbitrap) to produce a DIA chromatogram spectral library which was searched against the UniProt_human database. Experimental samples were blocked by replicate and randomized within each replicate. Injections of 2 μg of peptides and a 2-h HPLC gradient were employed. MS data were acquired using 12-*m*/*z* windows (staggered; 120k resolution for precursor scans, 30k for product ion scans) and searched against the chromatogram library. Scaffold DIA (v1.3.1; Proteome Software) was used for all DIA data processing. The pathway analysis was conducted with Reactome Pathway Database (https://reactome.org/) on differentially expressed proteins, focusing on the group that exhibited ≥ 1.5-fold change comparing mono and combination therapies to vehicle.

### Immunohistochemistry and γH2AX analysis

Immunohistochemical studies were performed as described previously [23]. For the immunohistochemical studies, tissue sections were blocked in 5% normal goat serum followed by overnight incubation with Ki-67 (1:100) primary antibody and subsequent secondary antibody incubation for 30 min at room temperature. Immunoreactivity was visualized by using the DAB substrate and counterstained with hematoxylin (Vector Lab, Inc.). Percent of Ki-67-positive proliferating cells was calculated in five randomly selected microscopic fields. For γH2AX analysis, MCF-7 cells were cultured on glass coverslips and treated with vehicle, ERX-11, palbociclib, or combination for 4 h. Cells were fixed in 3.7% paraformaldehyde followed by permeabilization with 0.1% TritonX-100 for 10 min. Cells were then stained with γH2AX antibody, and the fluorescence was analyzed by microscopy.

### Statistical analyses

Statistical differences between the groups were analyzed with either a *t* test or ANOVA as appropriate using GraphPad Prism 6 software. All the data represented in plots are shown as means ± SE. A value of *p* < 0.05 was considered statistically significant.

## Results

### ERX-11 and CDK4/6 inhibitors act synergistically to reduce the growth of therapy-sensitive and therapy-resistant BCa cells

**Fig. 1:**
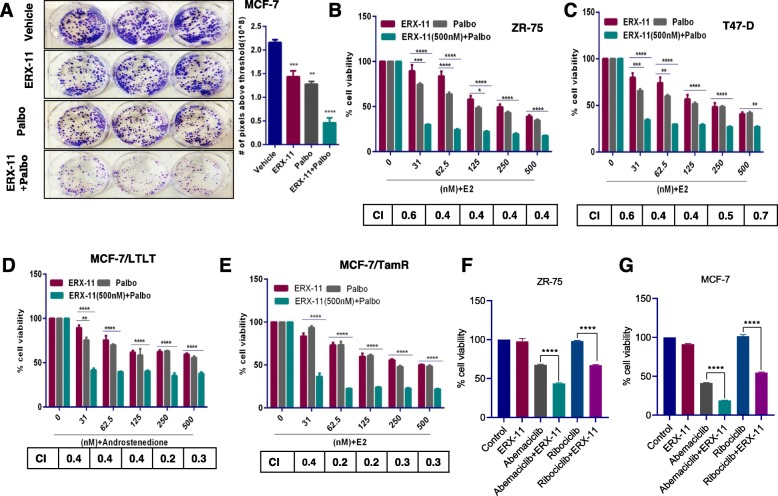
ERX-11 and palbociclib synergistically reduce the growth of ER+ and endocrine therapy-resistant BCa cells. **a** Equal numbers of MCF-7 cells were treated with ERX-11 (250 nM), palbociclib (50 nM), or in combination, and clonogenic (survival) assays were performed after 14 days. ZR-75 (**b**), T-47D (**c**), MCF-7/LTLT (**d**), and MCF-7/TamR (**e**) cells were stimulated with E2 (10^−8^ M) or androstenedione (10 ^−8^ M) for 7 days in the presence or absence of ERX-11 (0.5 μM), palbociclib (0.5 μM), or in combination, and the cell viability was measured by MTT assay. ZR-75 (**f**) and MCF-7 (**g**) cells were stimulated with E2 (10^−^8 M) for 7 days in the presence or absence of ERX-11 (31 nM), abemaciclib (31 nM), ribociclib (31 nM), or combination, and the cell viability was measured by MTT assay. The combination index (CI) was determined by the Chou-Talalay method, CI < 1 indicates synergism (*p < 0.05, **p < 0.01, ***p < 0.001, ****p < 0.0001)

Our recent studies demonstrated that ERX-11 had anti-proliferative activity on therapy-resistant ER+ BCa cells, although at 500 nM concentrations [23]. Since therapy-resistant BCa cells often exhibit deregulation of both CDK4/6 and ER coregulator-driven pathways, we hypothesized that the combination of ERX-11 and CDK4/6 inhibitor would more effectively block BCa cell proliferation. Colony formation assays visually demonstrated that the combination was more effective in reducing the colony formation of BCa cells compared to either ERX-11 or palbociclib monotherapy (Fig. 1a).

Therapy-sensitive (ZR-75, T-47D) and endocrine therapy-resistant (MCF-7/TamR, MCF-7/LTLT) BCa cells were treated with ERX-11 or the CDK4/6 inhibitor, palbociclib, or with the combination. MTT assays showed that combination therapy was more effective in reducing the cell viability compared to either ERX-11 or palbociclib alone (Fig. 1b–e). The combination index (CI) studies using the Chou-Talalay method [28] showed that the CI value was less than 1 in all the four BCa model cells tested and confirmed that the combination therapy was synergistic. In contrast, the combination of ERX-11 with other therapeutic agents like gemcitabine, cisplatin, or paclitaxel did not show any additive or synergistic effect (Additional file 1: Figure S1). To further test whether palbociclib enhances the efficacy of ERX-11 by lowering its IC50, we conducted a dose-response study with ERX-11 keeping the constant concentration of palbociclib. The results showed that palbociclib significantly enhanced the potency of ERX-11 and lowered the IC50 of ERX-11 in all five model cells (Additional file 1: Figure S2).

We then tested the utility of combination therapy using two additional CDK4/6 inhibitors currently in clinical trials/treatment including abemaciclib and ribociclib. The results from these studies showed that similar to palbociclib, both abemaciclib and ribociclib act synergistically with ERX-11 (Fig. 1f, g). We also confirmed the utility of ERX-11 combination therapy using three different CDK4/6 inhibitors in colony formation assays. Results showed that the ERX-11+CDK4/6 inhibitor combination was more effective in reducing the colony formation of BCa cells compared to either ERX-11, palbociclib, abemaciclib, or ribociclib monotherapy (Additional file 1: Figure S3).

**Fig. 2:**
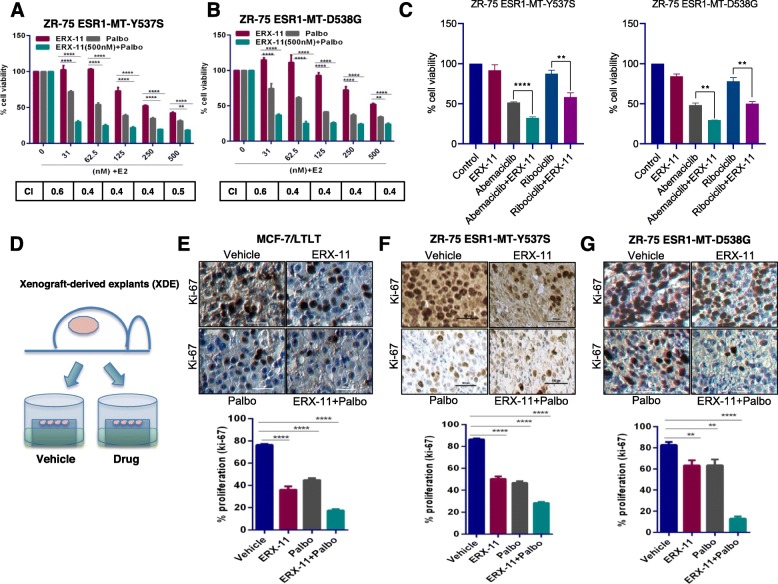
ERX-11 enhances the efficacy of palbociclib in reducing the growth of ER-MT and CDK4/6 inhibitor therapy-resistant BCa cells. ZR-75-ESR1-MT-Y537S (**a**) and ZR-75-ESR1-MT-D538G (**b**) cells were stimulated with E2 (10^−8^ M) for 7 days in the presence or absence of ERX-11 (0.5 μM), palbociclib (0.5 μM), or in combination, and the cell viability was measured by MTT assay. **c** ZR-75-ESR1-MT-Y537S and ZR-75-ESR1-MT-D538G cells were stimulated with E2 (10^-8^ M) for 7 days in the presence or absence of ERX-11 (31 nM), abemaciclib (31 nM), ribociclib (31 nM), or in combination, and the cell viability was measured by MTT assay. **d** Schematic representation of xenograft-derived explant (XDE) assay. The effect of ERX-11, palbociclib, and combination therapy on the proliferation of MCF-7/LTLT (**e**), ZR-75-ESR1-MT-Y537S (**f**), and ZR-75-ESR1-MT-D538G (**g**) tumors was measured by Ki-67 staining. The combination index (CI) was determined by the Chou-Talalay method, CI < 1 indicates synergism (**p < 0.01, ****p < 0.0001)

We next treated ER-mutant model cells (ZR-75-ESR1-MT-Y537S, ZR-75-ESR1-MT-D538G) with ERX-11 or the CDK4/6 inhibitors, or with the combination. The results showed that the combination therapy has a synergistic effect in these ER-mutant model cells (Fig. 2a, b, c). We then tested whether ERX-11 has utility in treating MCF-7/RR cells that have acquired resistance to the CDK4/6 inhibitor, ribociclib [24]. We noted that both MCF-7 and MCF-7/RR were responsive to ERX-11. As expected, due to the acquired resistance of MCF-7/RR cells to ribociclib, the combination of ERX-11+ribociclib was not additive in MCF-7/RR cells; however, they are sensitive to ERX-11 therapy (Additional file 1: Figure S4A). Furthermore, MCF-7/RR exhibited resistance to antiestrogen fulvestrant (ICI); however, ERX-11 was able to reduce the cell viability of MCF-7/RR (Additional file 1: Figure S4B, C). Collectively, these results suggest that ERX-11-targeted molecular vulnerabilities enhance palbociclib efficacy, and combination therapy will have utility in treating therapy-sensitive and therapy-resistant BCa.

### ERX-11 and CDK4/6 inhibitor combination is effective in reducing the proliferation of xenograft breast tumor explants

We then evaluated the effect of ERX-11 and palbociclib combination therapy using ex vivo cultures of xenograft tumors (xenograft-derived explants (XDE)) of two prevalent ER mutants (ZR-75-ESR1-MT-Y537S, ZR-75-ESR1-MT-D538G) [29–32] and letrozole-resistant MCF-7/LTLT tumors and examined the expression of Ki-67 as a marker of proliferation. The results showed that ERX-11 and palbociclib combination is more efficient in reducing the expression of Ki-67 in all three XDEs including those from MCF-7/LTLT, ZR-75-ESR1-MT-Y537S, and ESR1-MT-D538G (Fig. 2d–g). These results validate our cell line data and suggest that the combination of ERX-11 and palbociclib can inhibit the growth of ER+ human breast tumors and therapy-resistant xenograft tumors ex vivo.

### Combination of ERX-11 with CDK4/6 inhibitor promoted tumor regression in preclinical xenograft models of endocrine resistance

**Fig. 3:**
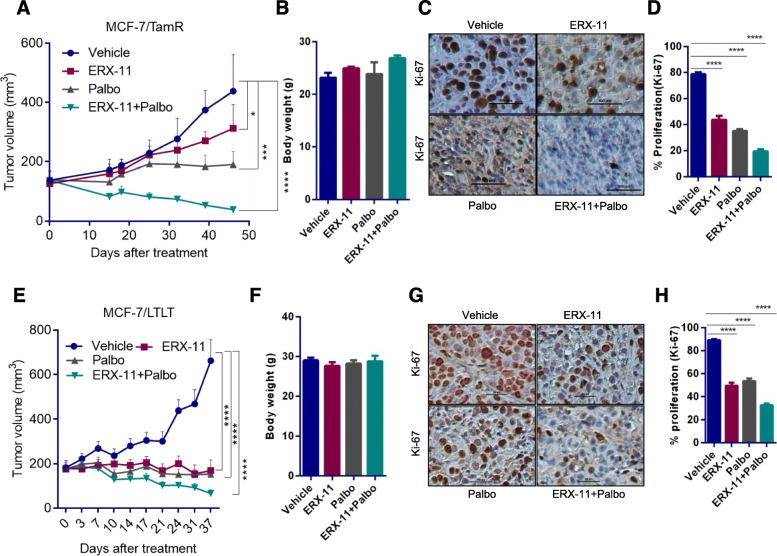
ERX-11 enhances palbociclib ability to reduce tumor growth in preclinical models of endocrine therapy resistance. **a**–**d** Following implantation and growth of ER+ tamoxifen-resistant xenografts (*n* = 4 tumors/group), mice were treated with vehicle, ERX-11 (10 mg/kg/day), palbociclib (50 mg/kg/day), or in combination. Tumor volume (**a**), body weight (**b**), and Ki-67 status of vehicle and drug-treated tumors were shown (**c**, **d**). Following implantation and growth of ER+ letrozole resistant xenografts (*n* = 7 tumors/group), mice were treated with vehicle or ERX-11(10 mg/kg/day), palbociclib (50 mg/kg/day), or in combination. Tumor volume (**e**), body weight (**f**), and Ki-67 status of vehicle and drug-treated tumors were shown (**g**, **h**) (*p < 0.05, ***p < 0.001, ****p < 0.0001)

In vivo efficacy of ERX-11 and palbociclib combination therapy was evaluated using MCF-7/TamR xenograft model that exhibits resistance to tamoxifen treatment. MCF-7/TamR xenografts (*n* = 4 tumors/group) containing nude mice were randomized to feed via oral gavage 5 days/week with 10 mg/kg ERX-11 or 50 mg/kg palbociclib or combination. Combination treatment resulted in significantly lower tumor volume and smaller tumor size compared to monotherapy of ERX-11 or palbociclib (Fig. 3a). Mice treated with ERX-11 and palbociclib combination exhibited no overt signs of toxicity, and body weight was not affected (Fig. 3b) compared to vehicle or monotherapy. Furthermore, ERX-11- and palbociclib-treated tumors exhibited less proliferation (Ki-67 staining) compared to vehicle or monotherapy *(*Fig. 3c, d). We also confirmed the efficacy of combination therapy using MCF-7/LTLT xenograft tumors that exhibit resistance to letrozole (*n* = 7 tumors/group). Results showed that ERX-11 and palbociclib combination is more efficient in reducing the letrozole-resistant tumor volume and proliferation (Fig. 3e–h). Collectively, these data indicate that ERX-11 and palbociclib combination is more potent in reducing the growth of endocrine therapy-resistant breast tumors in vivo.

### ERX-11 and CDK4/6 inhibitor combination is effective in reducing the proliferation of primary patient-derived breast tumor explants

**Fig. 4:**
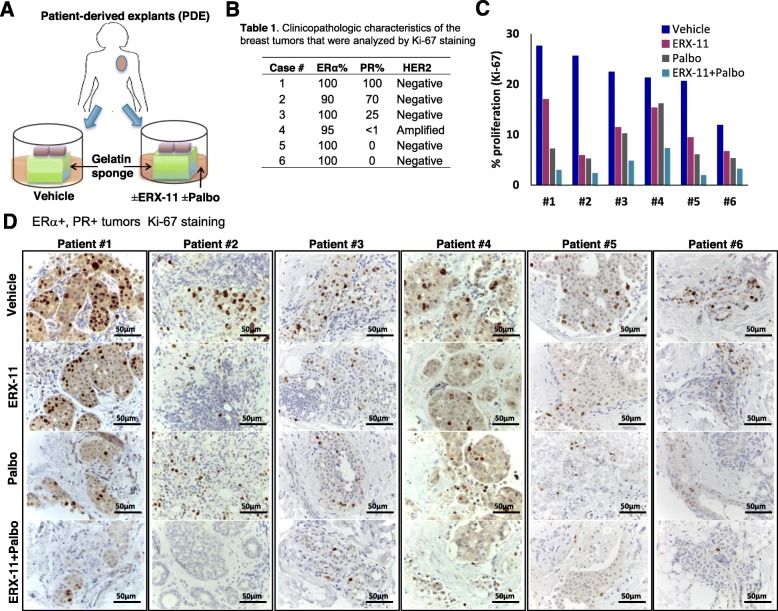
ERX-11 enhances palbociclib ability to reduce proliferation in patient-derived tumor explants. Schematic representation of patient-derived explant (PDE) model is shown (**a**). Six PDEs (**b**) were treated with vehicle, ERX-11, palbociclib, or in combination for 72 h. Effect of ERX-11, palbociclib, or combination therapy on Ki-67 expression in ER+ tumors is shown (**c**, **d**)

To test the efficacy of ERX-11 and palbociclib combination therapy on primary breast specimens, we have used patient-derived explants (PDE) of primary breast tumors that allow for the evaluation of drugs on breast tumors while maintaining their native tissue architecture [33, 34]. Surgically extirpated de-identified breast tissues are sliced into small pieces and grown ex vivo on a gelatin sponge and treated with either vehicle, ERX-11, palbociclib, or ERX-11+palbociclib (Fig. 4a, b). Combination therapy of ERX-11 and palbociclib potently reduced the proliferation (Ki-67 staining) in PDEs obtained from 6/6 patients compared to untreated vehicle, ERX-11, or palbocliclib monotherapy (Fig. 4c, d).

### ERX-11 and CDK4/6 inhibitor treatment alters the interactions between ER and coregulators

**Fig. 5:**
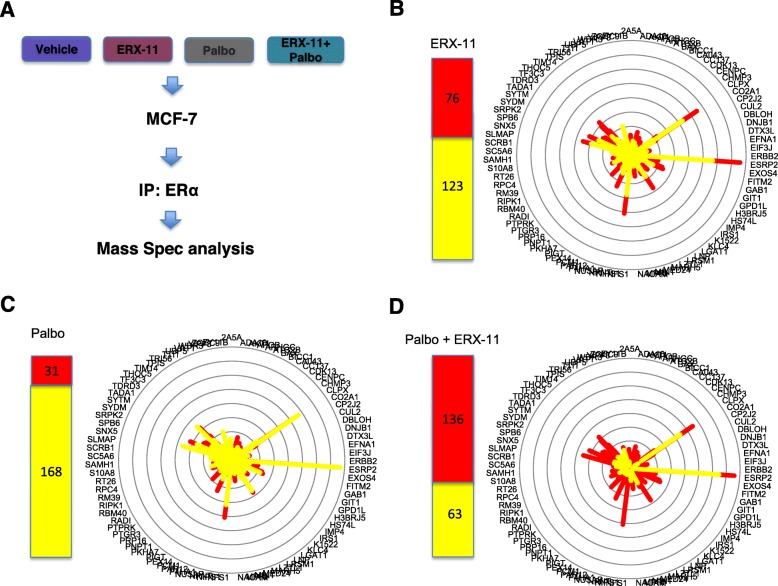
ERX-11+palbociclib treatment alters ER interactions with coregulators. **a**–**d** MCF-7 cells were treated with E2 (10^−8^ M) in the presence or absence of ERX-11 (10 μM), palbociclib (0.25 μM), or ERX-11+palbociclib for 2 h. Cell lysates from treated cells were subjected to immunoprecipitation with ERα antibody. The immunoprecipitates were analyzed by mass spectrometry

Since ERX-11 blocks ER interactions with coregulators, we examined the effect of the combination therapy on the interactions between ER with coregulators. Using an unbiased immunoprecipitation mass spectrometry approach, we showed that the combination therapy significantly disrupted the interactions between ER and a larger number of ER-binding coregulators than either monotherapy or vehicle controls. Indeed, the combination disrupted 136/199 coregulator interactions with ER in MCF-7 cells compared to 31 and 76 coregulators blocked by palbociclib or ERX-11 alone (Fig. 5a–d, Additional file 1: Figure S5). We also confirmed the ability of ERX-11+palbo combination therapy to potently disrupt ERα interaction with coregulators using an immunoprecipitation experiment that examined the interaction of two well-known ERα coregulators SRC1 and SRC3. The results confirmed that treatment of combination therapy potently disrupted the interaction of ERα with both SRC1 and SRC3 compared to monotherapy (Additional file 1: Figure S6A). Collectively, these studies suggested that coregulator interaction with ER was globally affected by the ERX-11 and palbociclib combination therapy.

### ERX-11 did not enhance CDK4/6 inhibitor-mediated G0–G1 cell cycle arrest

Effects of ERX-11 and palbociclib combination therapy on cell cycle progression were investigated using cell cycle analyses. As expected from the inhibition of CDK4/6 activity, both MCF-7 and MCF-7/TamR cells showed an increase in the G0–G1 phase of the cell cycle. However, combination therapy did not promote any further increase than palbociclib as a monotherapy (Additional file 1: Figure S6B). Western blot analysis also showed a lack of additive effect of combination therapy on the levels of various cell cycle proteins including cyclin D1, p21, and pERK1/2 (Additional file 1: Figure S6C). Collectively, these findings suggest that the higher efficacy seen in ERX-11 and palbociclib combination therapy is not due to cell cycle alterations.

### Global mass spectrometry-based analyses identified unique pathways modulated by combination therapy

**Fig. 6:**
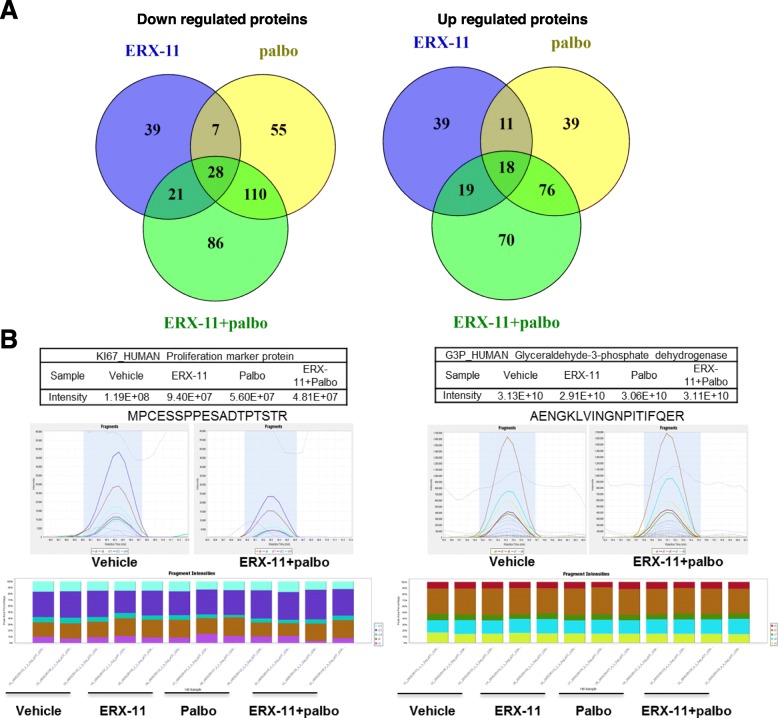
Mass spectrometry-based DIA analyses identified unique pathways modulated by ERX-11+palbociclib combination therapy. MCF-7/TamR cells were treated with E2 (10^−8^ M) in the presence or absence of ERX-11(1 μM), palbociclib (0.25 μM), or ERX-11+palbociclib for 72 h. **a** Venn diagram showing differentially expressed proteins ≥ 1.5-fold difference (either up or down) among the groups. **b** Scaffold DIA results for GAPDH and Ki-67 (protein data in the table and representative fragment intensities displayed underneath)

To understand the mechanism of synergy between ERX-11 and palbociclib, we used data-independent acquisition (DIA) mass spectrometry analyses using whole-cell lysates of MCF-7/TamR cells treated with vehicle, ERX-11, palbociclib, or combination of ERX-11 and palbociclib in 3 biological replicates. DIA analyses resulted in the identification of 4449 proteins (4442 quantified with 2 or more peptides). Proteins exhibiting ≥ 1.5-fold difference (either up or down) in relative quantity after treatment with ERX-11, palbociclib, or combination of ERX-11+palbociclib compared to vehicle are shown in the Venn diagram (Fig. 6a). Importantly, in agreement with immunohistochemical results from PDE, XDE, and xenografts, DIA analysis showed significant downregulation of the proliferation marker Ki-67 in response to combination therapy compared to monotherapy or vehicle. In contrast, no effect on the expression of housekeeping genes such as GAPDH was observed by either mono or combination therapy (Fig. 6b). A comparison was made of relative protein abundance between vehicle and combination therapy, and proteins that were downregulated or upregulated ≥ 1.5-fold were subjected to pathway analysis using Reactome (https://reactome.org/). Selected pathways are shown in Additional file 1: Figure S7*.* The unique pathways modulated by the combination therapy include E2F signaling, RNA metabolism, translation, DNA damage, DNA repair, apoptosis, and endoplasmic reticulum stress. Representative results from the DIA analysis of selected proteins involved are shown (Additional file 1: Figure S8). We have utilized a widely used γ-H2AX foci assay that indicates the presence of a double-strand break (DSB) and foci disappearance indicate repair of the DNA damage. The results confirmed an increase in the accumulation of γ-H2AX foci in combination therapy-treated cells compared to monotherapy of ERX-11 or palbociclib or untreated control (Additional file 1: Figure S9).

### ERX-11 and palbociclib treatment decrease the levels of E2F1, ERα, and ER-coregulators

**Fig. 7:**
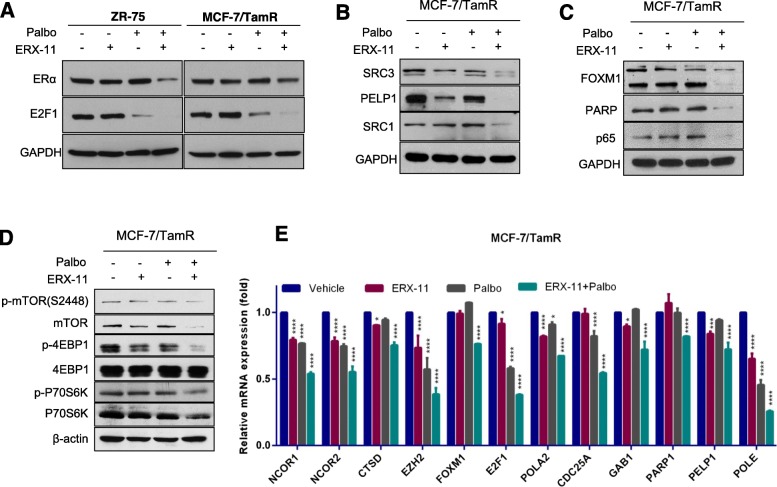
ERX-11 enhances palbociclib-mediated alterations in the levels of ER, E2F, and their downstream targets. **a** ZR-75 and MCF-7/TamR cells were treated with E2 in the presence or absence of ERX-11, palbociclib, or ERX-11+palbociclib, and the status of ERα and E2F1 was analyzed by western blotting. **b** MCF-7/TamR cells were treated with E2 in the presence or absence of ERX-11, palbociclib, or ERX-11+palbociclib, and the status of coregulators was analyzed by western blotting. **c** MCF-7/TamR cells were treated with E2 in the presence or absence of ERX-11, palbociclib, or ERX-11+palbociclib, and the status of FOXM1, PARP, and p65 was analyzed by western blotting. **d** MCF-7/TamR cells were treated with E2 in the presence or absence of ERX-11, palbociclib, or ERX-11+palbociclib, and the status of mTOR signaling components were analyzed by western blotting. **e** MCF-7/TamR cells were treated in the presence or absence of ERX-11, palbociclib, or ERX-11+palbociclib for 6 h, and RT-qPCR analysis was conducted for indicated genes (*p < 0.05, ***p < 0.001, ****p < 0.0001)

Western blot analysis using two BCa models also confirmed the downregulation of ERα and E2F1 expression upon treatment with combination therapy compared to monotherapy *(*Fig. 7a). Further, western blot analysis revealed that the combination therapy reduced the levels of several oncogenic ER coregulators including SRC3, PELP1, and SRC1 (Fig. 7b). Moreover, western blot analyses confirmed the downregulation of DNA repair proteins identified in DIA analyses including FOXM1, PARP1, and NF-κB subunit p65 following combination therapy (Fig. 7c). Since global proteomic analysis results (Fig. 6) suggested the downregulation of pathways involved in protein translation, we confirmed whether ERX-11 and palbociclib therapy alters the mTOR signaling axis. The results showed that ERX-11 and palbociclib therapy substantially reduced the expression of several components of mTOR signaling including mTOR, 4EBP1, and p70S6K compared to monotherapy (Fig. 7d). The RT-qPCR analysis confirmed the significant downregulation of several genes regulated by the E2F1 and ER pathways by combination therapy (Fig. 7e). These data indicate that the downregulation of E2F1, ERα, and ER binding proteins may contribute functionally to the alteration in the ER coregulator binding profile and consequently to ER activity. Taken together, these results along with unbiased global mass spectrometry-based analyses (Fig. 6) indicate that the effect of the combination therapy may be related to the dramatic decrease of the expression of a number of ER coregulators, ER, and E2F downstream signaling proteins (Additional file 1: Figure S10).

## Discussion

The majority of breast cancer is ER+. However, both de novo and acquired therapy resistance limit the utility of ER-targeted therapy using AIs and AEs. Emerging evidence implicate ER signaling is intact in many therapy-resistant tumors, and ER interaction with critical coregulator proteins mediates ER signaling in these therapy-resistant tumors. While AIs and AEs may disrupt some of these ER-coregulator interactions, their ability to target these interactions is limited in therapy-resistant cells. Recently, we developed ERX-11, a small molecule that targets ER at a unique site, that functions as an ER-coregulator modulator and has good efficacy in treating therapy-resistant BCa [23]. Since extensive crosstalk occurs with cell cycle machinery and ER coregulators, we reasoned that combination treatments that block ER-coregulator signaling and CDK4/6 signaling may have better therapeutic utility. Using multiple in vitro cell-based assays, ex vivo PDE, xenograft-derived MT-ER explants, and preclinical xenograft models of endocrine resistance, this study provided evidence that combination therapy of ERX-11 and palbociclib is very efficient in blocking the growth of therapy-resistant BCa.

Approximately 70% of BCa are dependent on ER signaling for growth. ER signaling modulates multiple pathways including upregulation of cyclin D1 which upregulate CDK4/6 activity [35, 36]. Cyclin D1 amplification occurs in 58% of luminal B cancers and 29% of luminal A cancers [20]. Increased cyclin D1 potentiates ER transcription in an autocrine manner further potentiating ER-mediated cell proliferation. This scientific premise formed the basis for combining endocrine therapy with CDK4/6 inhibitors to overcome ER therapy resistance. Recently, the FDA approved CDK4/6 inhibitors for clinical use in the metastatic setting in combination with letrozole or with fulvestrant [37]. All three CDK4/6 inhibitors (palbociclib, ribociclib, abemaciclib) showed prolongation of progression-free survival (PFS) over ER targeted therapy. Unfortunately, most patients eventually acquire resistance to CDK4/6 inhibitors, and disease progression occurs. Preclinical studies identified several mechanisms that contribute to resistance including acquired mutation of RB1, amplification of cyclin E, amplification of CDK6 or suppression of CDK2 inhibitors (e.g., p27kip1 or p21cip1), and alterations in the PI3K/mTOR pathway [38]. Therefore, combining CDK4/6 inhibitor therapy with an effective ER target therapy will further maximize CDK4/6 inhibitors utility. Our results support the utility of ERX-11 in extending the efficacy of CDK4/6 inhibitors (palbociclib, ribociclib, abemaciclib) for treating therapy-resistant BCa. Further, ERX-11 was also effective in reducing the growth of CDK4/6 inhibitor-resistant models.

ER-coregulators play an important role in ER mediated cell proliferation. For example, SRC3 directly interacts with E2F1, is recruited to E2F target gene promoters, and stimulates the transcription of a subset of E2F-responsive genes that are associated with the G1/S transition [39]. Knockdown of SRC2 or SRC3 coactivators decreases the E2-induced progression from G1 to S [40]. ER coregulator, PELP1, is a novel substrate of CDKs, phosphorylated by the CDK4/cyclin D1, couples ER signaling to the E2F axis, and CDK phosphorylation plays a key role in the PELP1 oncogenic functions [18]. ER-coregulator CARM1 is required for the E2-mediated activation of E2F1 and for the induction of E2F1 target genes [41]. Since crosstalk occurs between ER and E2F1 signaling, blockage of ER-coregulator signaling further potentiates the inhibition of E2F signaling mediated by palbociclib. Our results showed that the combination of ERX-11 and palbociclib is more efficient in decreasing E2F1 protein levels compared to monotherapy of ERX-11 or palbociclib. Further, our studies demonstrated a higher efficacy of combination therapy in altering both ER and E2F signaling. These results suggest that the co-therapeutic targeting of ERX-11 with palbociclib is beneficial in treating therapy-resistant BCa.

Alterations in the concentration or activity of selective coregulators enable ER signaling from AE-ER complexes, effectively converting the antagonist to an agonist [15, 16]. Over one third (38%) of ER coregulators identified in BCa are over-expressed [4–6], such as SRC3 [7, 8], SRC2 [9], and PELP1 [10]. The unbiased IP mass spectrometry analyses suggest that ERX-11 and palbociclib combination is more efficient in blocking the interactions of ER with multiple coregulators. Several mechanisms might have contributed to the increased efficacy of ERX-11 and palbociclib combination in the disruption of the ER interactome. Since ER and its coregulator expression and functions are modulated throughout the cell cycle via phosphorylation by CDKs, CDK4/6 inhibition could indirectly affect ER interactions by altering ER phosphorylation, coregulator phosphorylation, and their expression by altering ER and E2F transcription factors. In support of this, RT-qPCR analyses showed that ERX-11 and palbociclib combination therapy is more efficient in suppressing key pathways including ER and E2F. Western blot analyses showed that combination therapy has the potential to reduce the expression levels of several coregulators. Thus, the more comprehensive disruption of ER coregulators by the combination of ERX-11 and CDK4/6 inhibitors represents an exciting approach in therapy-resistant BCa.

Mutations in ER are rare in untreated patients. However, recent studies revealed that breast tumors acquire mutations in the ER ligand-binding domains (L536 N, Y537S, Y537N, and D538G) that facilitate the constitutive activity of these mutant ER (MT-ER) in the absence of ligand [29–32]. Tumors with MT-ER interact with oncogenic coregulators to drive ER-dependent transcriptional programs and proliferation and are poorly responsive to AEs and AIs [17, 29–32]. Recent studies reported encouraging activity of fulvestrant and palbociclib combination therapy against MT-ER cancers; however, it also suggested that combination treatment with palbociclib and letrozole does not prevent the selection of ER mutations [42]. Therefore, endocrine agents that are effective against MT-ER will have more efficacy in treating advanced BCa. ERX-11 interacts directly with MT-ER and efficiently blocks their oncogenic signaling [23]. Our results showed that ERX-11 significantly enhanced the efficacy of palbociclib in reducing the growth of MT-ER expressing cells. Importantly, using ex vivo culture of xenograft-derived MT-ER tumor tissues, we demonstrated that ERX-11 and palbociclib combination therapy is effective in limiting the proliferation of ER-MT-driven tumors.

E2F-mediated gene transcription plays a critical role in DNA damage response and repair [43]. CDK4/6 inhibitors are known to cause suppression of poly (ADP-ribose) polymerase 1 (PARP1) transcription [44], and PARP1 protein is involved in several mechanisms of DNA damage repair. In this study using DIA based whole-cell lysate mass spectrometry analysis, we found that ERX-11 and palbociclib combination therapy significantly downregulated the DNA damage response and repair pathways compared to monotherapy [45]. Independent biochemical assays also confirmed the downregulation of FOXM1 and PARP1 proteins. Since FOXM1 drives the transcription of genes for DNA damage sensors, mediators, signal transducers, and effectors [46], a decrease in FOXM1 reduces the DNA damage repair gene expression network and this might have contributed to enhanced sensitization of ERX-11 and palbociclib combination therapy.

## Conclusions

Our findings suggested that combination therapy of ERX-11 and CDK4/6 inhibitors may be a promising therapeutic strategy for therapy-resistant BCa. Mechanistic studies revealed that ERX-11 and CDK4/6 inhibitor combination therapy potency is mediated by (1) higher efficacy in altering both ER and E2F signaling, (2) more comprehensive disruption of ER coregulators, and (3) decreased DNA damage repair gene expression network. Since ERX-11 is well-tolerated with fewer side effects and has activity against BCa resistant to both endocrine therapy and CDK4/6 inhibitors, it can be readily extended to clinical use as a therapeutic to enhance the utility of CDK4/6 inhibitors.

## Supplementary information

**Additional file 1: Figure S1.** (A) MCF-7 or (B) T-47D cells were stimulated with E2 (10^-8^M) for 3 days in the presence or absence of 1 μM of ERX-11 or cisplatin or paclitaxel or gemcitabine or in combination and the cell viability was measured by Cell Titre-Glo Luminescent assay. **Figure S2.** ZR-75, T-47D, MCF-7/TamR, ZR-75-ESR1-MT-Y537S and ZR-75-ESR1-MT-D538G cells were stimulated with E2 (10^-8^M) for 7 days in the presence or absence of ERX-11 (0.5μM) or palbociclib (0.5μM) or in combination with indicated concentrations of ERX-11 and the cell viability was measured by MTT assay. **Figure S3.** Equal number of ZR-75, ZR-75-ESR1-MT-Y537S and ZR-75-ESR1-MT-D538G cells were plated and treated with ERX-11 (500nM) or palbociclib (50 nM) or abemaciclib (50nM) or ribociclib (50nM) or combination and clonogenic (survival) assays were performed after 14 days. **Figure S4.** (A) Parental MCF-7 or ribociclib resistant MCF-7/RR cells were stimulated with E2 (10^-8M^) for 5 days in the presence or absence of ERX-11 (1, 5, 10 μM) or ribociclib (1 μM) or in combination and the cell viability was measured by Cell Titer-Glo Luminescent assay. (B) MCF-7 or (C) MCF-7/RR cells were treated with E2 (10^-8^M) for 5 days in the presence or absence of ERX-11 (1, 2, 5 μM) or ICI (0.2, 0.4, 1 μM). **Figure S10.** Schematic representation of model for mechanisms of ERX-11+palbociclib therapy.

## Data Availability

All data generated for this study are included within this article and in the supplementary information.

## Abbreviations

AE: Anti-estrogen
AI: Aromatase inhibitor
CDK: Cell cycle-dependent kinase
DIA: Data-independent acquisition
E2: Estrogen
ER: Estrogen receptor
ERX-11: Estrogen receptor coregulator binding modulator -11
ESR1: Estrogen receptor alpha
IHC: Immunohistochemistry
MCF-7/LTLTca: MCF7 cells resistant to letrozole
MCF-7/RR: MCF7 cells: resistant to ribociclib
MCF7-Tam: MCF7 cells resistant to tamoxifen
MT-ER: Estrogen receptor with mutation
PDE: Patient-derived explants
PELP1: Proline, glutamic acid, and leucine-rich protein-1
PI3K: Phosphotidyl inositol 3-kinase
RT-qPCR: Real-time quantitative PCR
SERD: Selective estrogen receptor degrader
SRC: Steroid receptor coregulator
XDE: Xenograft-derived explants
