# Supplementary material for: Estrogen receptor coregulator binding modulator (ERX-11) enhances the activity of CDK4/6 inhibitors against estrogen receptor-positive breast cancers
Source: Breast Cancer Res. 2019 Dec 26;21:150. doi: 10.1186/s13058-019-1227-8 (PMC6933697; doi:10.1186/s13058-019-1227-8)
Supplement: Supplementary file 1 — Additional file 1: Figure S1. (A) MCF-7 or (B) T-47D cells were stimulated with E2 (10-8M) for 3 days in the presence or absence of 1 μM of ERX-11 or cisplatin or paclitaxel or gemcitabine or in combination and the cell viability was measured by Cell Titre-Glo Luminescent assay. Figure S2. ZR-75, T-47D, MCF-7/TamR, ZR-75-ESR1-MT-Y537S and ZR-75-ESR1-MT-D538G cells were stimulated with E2 (10-8M) for 7 days in the presence or absence of ERX-11 (0.5μM) or palbociclib (0.5μM) or in combination with indicated concentrations of ERX-11 and the cell viability was measured by MTT assay. Figure S3. Equal number of ZR-75, ZR-75-ESR1-MT-Y537S and ZR-75-ESR1-MT-D538G cells were plated and treated with ERX-11 (500nM) or palbociclib (50 nM) or abemaciclib (50nM) or ribociclib (50nM) or combination and clonogenic (survival) assays were performed after 14 days. Figure S4. (A) Parental MCF-7 or ribociclib resistant MCF-7/RR cells were stimulated with E2 (10-8M) for 5 days in the presence or absence of ERX-11 (1, 5, 10 μM) or ribociclib (1 μM) or in combination and the cell viability was measured by Cell Titer-Glo Luminescent assay. (B) MCF-7 or (C) MCF-7/RR cells were treated with E2 (10-8M) for 5 days in the presence or absence of ERX-11 (1, 2, 5 μM) or ICI (0.2, 0.4, 1 μM). Figure S10. Schematic representation of model for mechanisms of ERX-11+palbociclib therapy. [file 13058_2019_1227_MOESM1_ESM.pptx]

## Slide 1
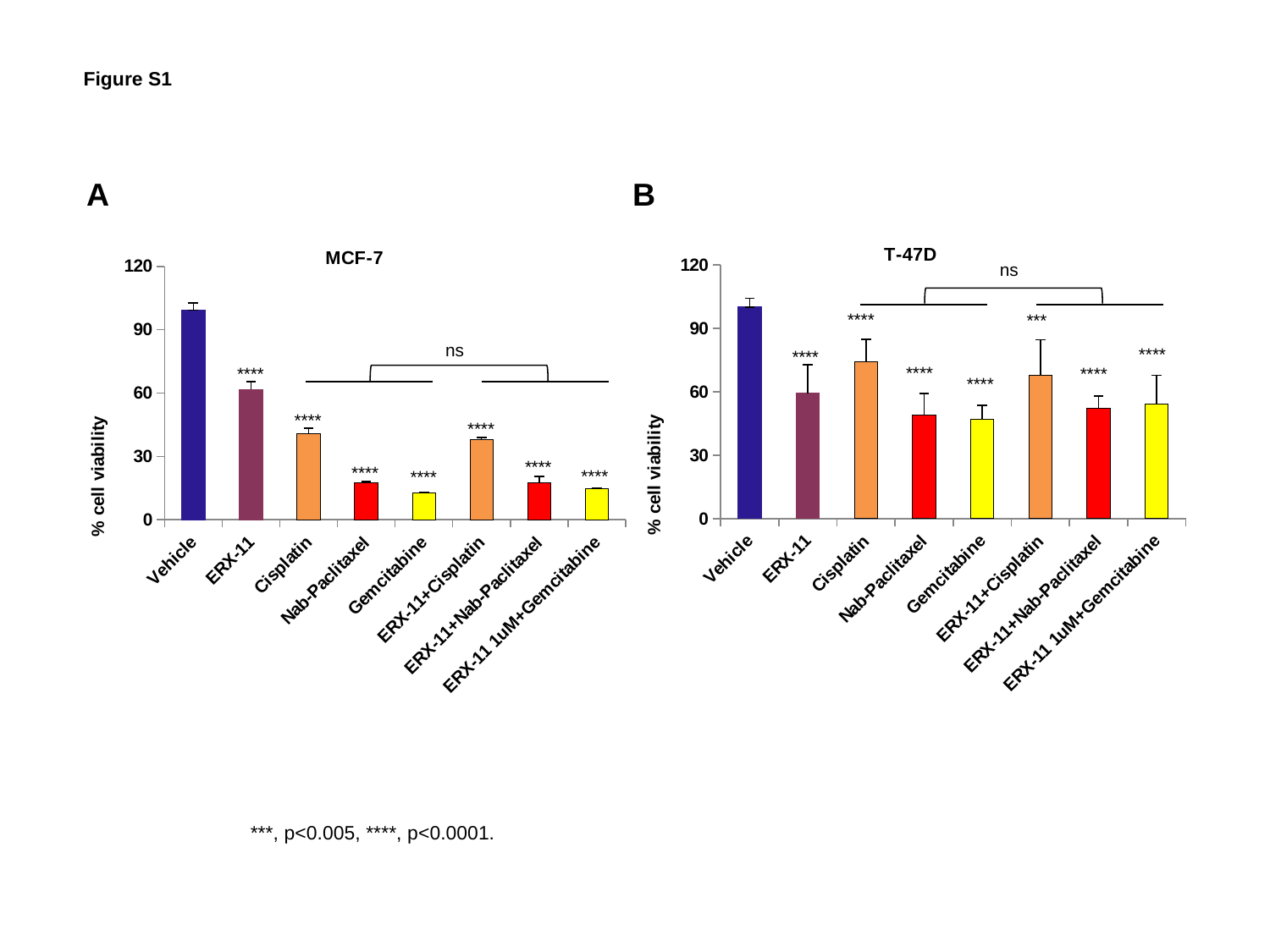

Figure S1
A
B
ns
### Chart: T-47D
| Category | T47D |
|---|---|
| Vehicle | 100.0 |
| ERX-11 | 59.23362592842674 |
| Cisplatin | 74.02400098213738 |
| Nab-Paclitaxel | 48.769259100116635 |
| Gemcitabine | 46.99066969492358 |
| ERX-11+Cisplatin | 67.78282487262906 |
| ERX-11+Nab-Paclitaxel | 52.042538825118164 |
| ERX-11 1uM+Gemcitabine | 54.24314038426126 |****
***
****
****
****
****
### Chart:
| Category | MCF-7 |
|---|---|
| Vehicle | 99.31815029970569 |
| ERX-11 | 61.77827942740035 |
| Cisplatin | 40.71913955960984 |
| Nab-Paclitaxel | 17.43763193984314 |
| Gemcitabine | 12.569533260397245 |
| ERX-11+Cisplatin | 38.015239533414494 |
| ERX-11+Nab-Paclitaxel | 17.594033622509517 |
| ERX-11 1uM+Gemcitabine | 14.67286623418648 |****
****
****
****
ns
***, p<0.005, ****, p<0.0001.

## Slide 2
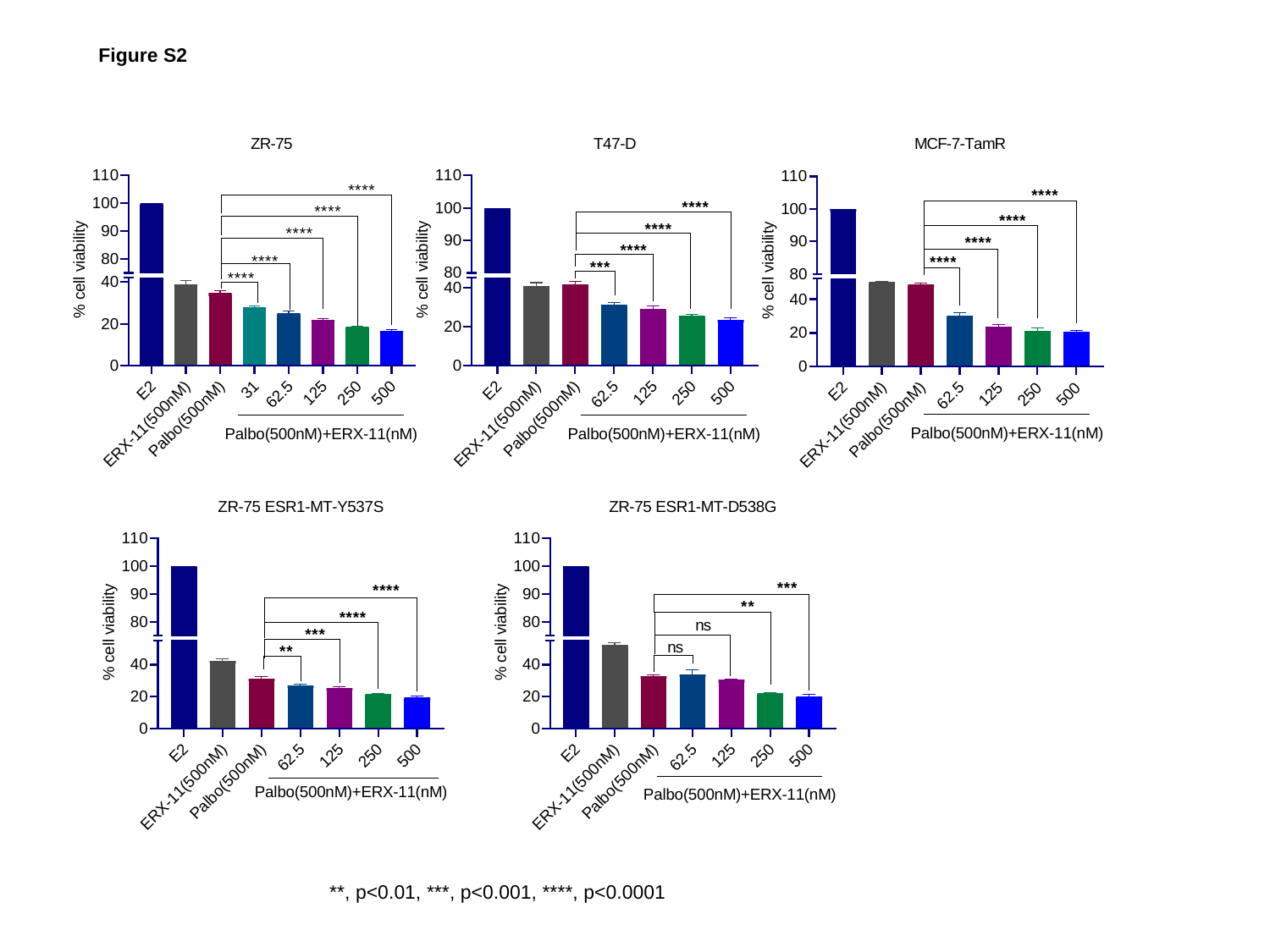

Figure S2
 **, p<0.01, ***, p<0.001, ****, p<0.0001

## Slide 3
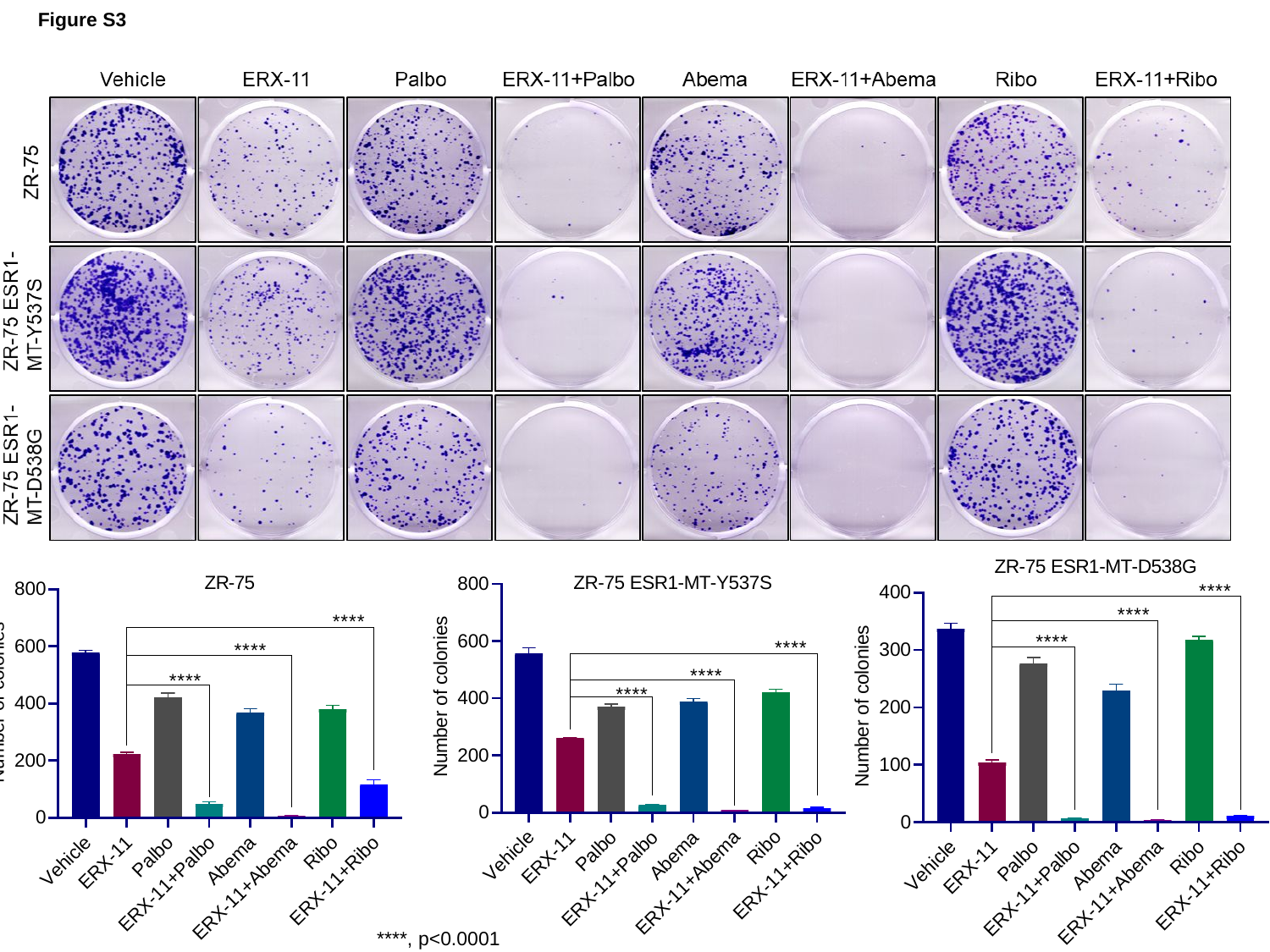

Figure S3
 ****, p<0.0001

## Slide 4
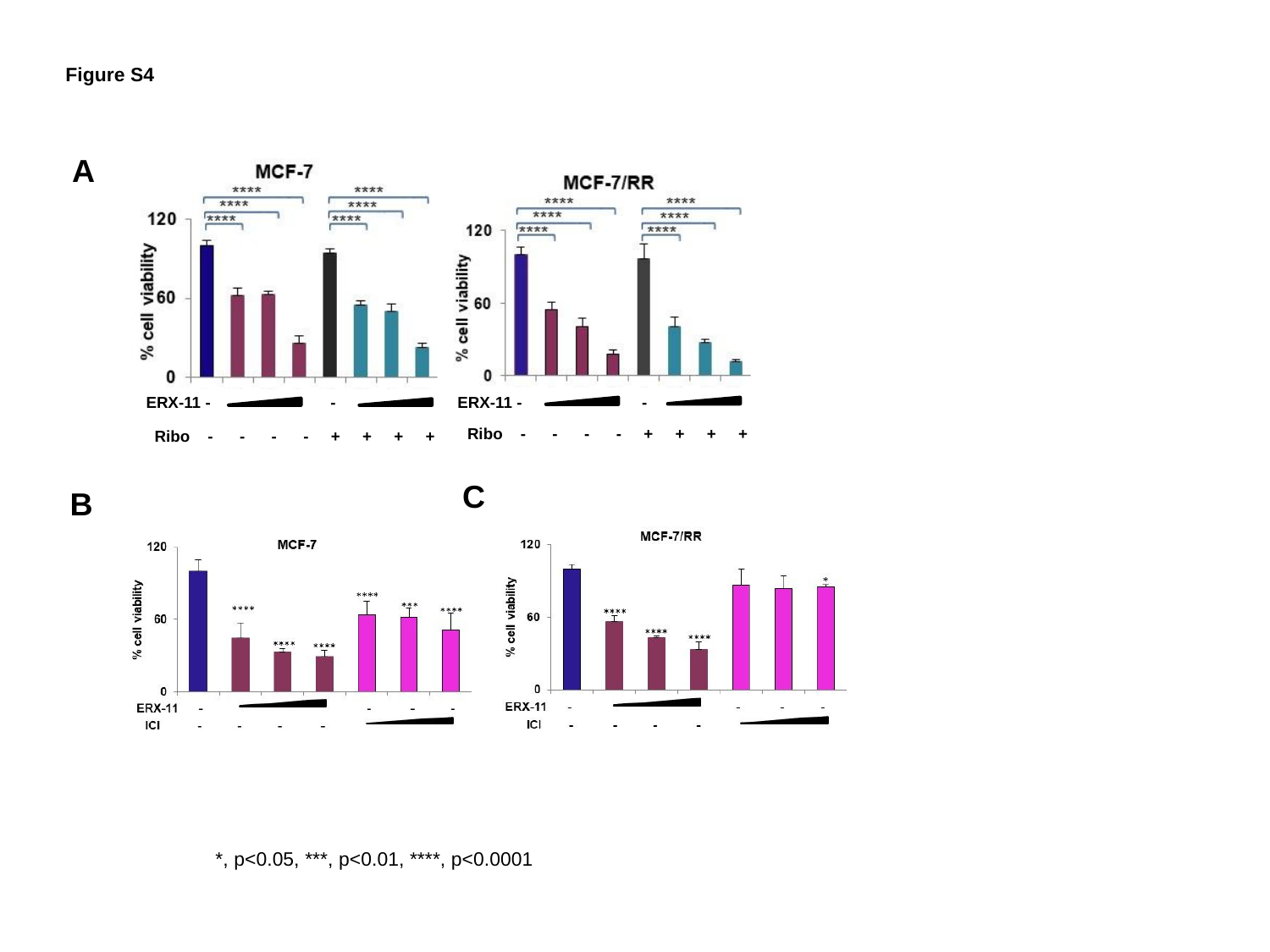

Figure S4
ERX-11 - -
Ribo - - - - + + + +
A
ERX-11 - -
Ribo - - - - + + + +
C
B
*, p<0.05, ***, p<0.01, ****, p<0.0001

## Slide 5
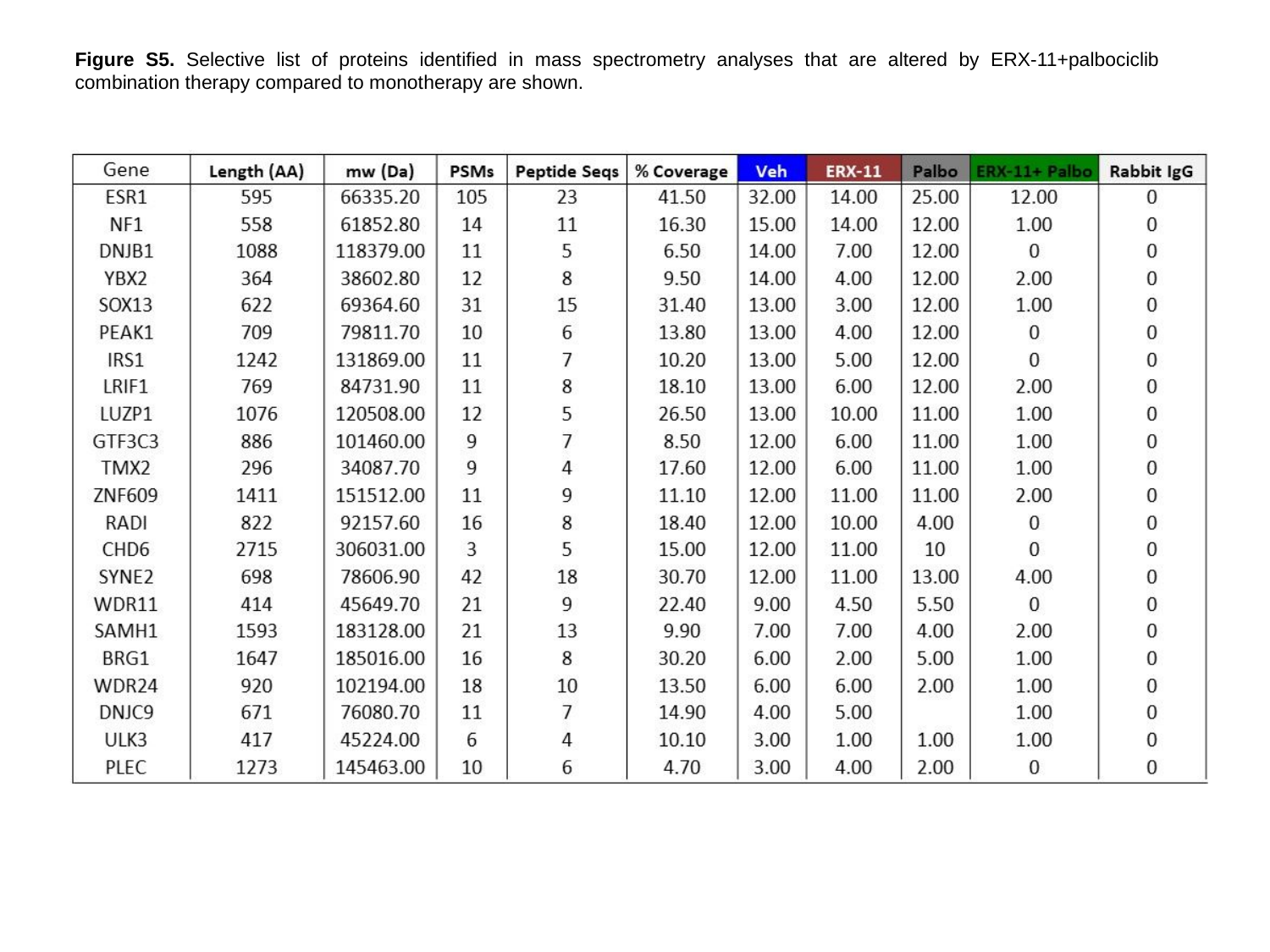

Figure S5. Selective list of proteins identified in mass spectrometry analyses that are altered by ERX-11+palbociclib combination therapy compared to monotherapy are shown.

## Slide 6
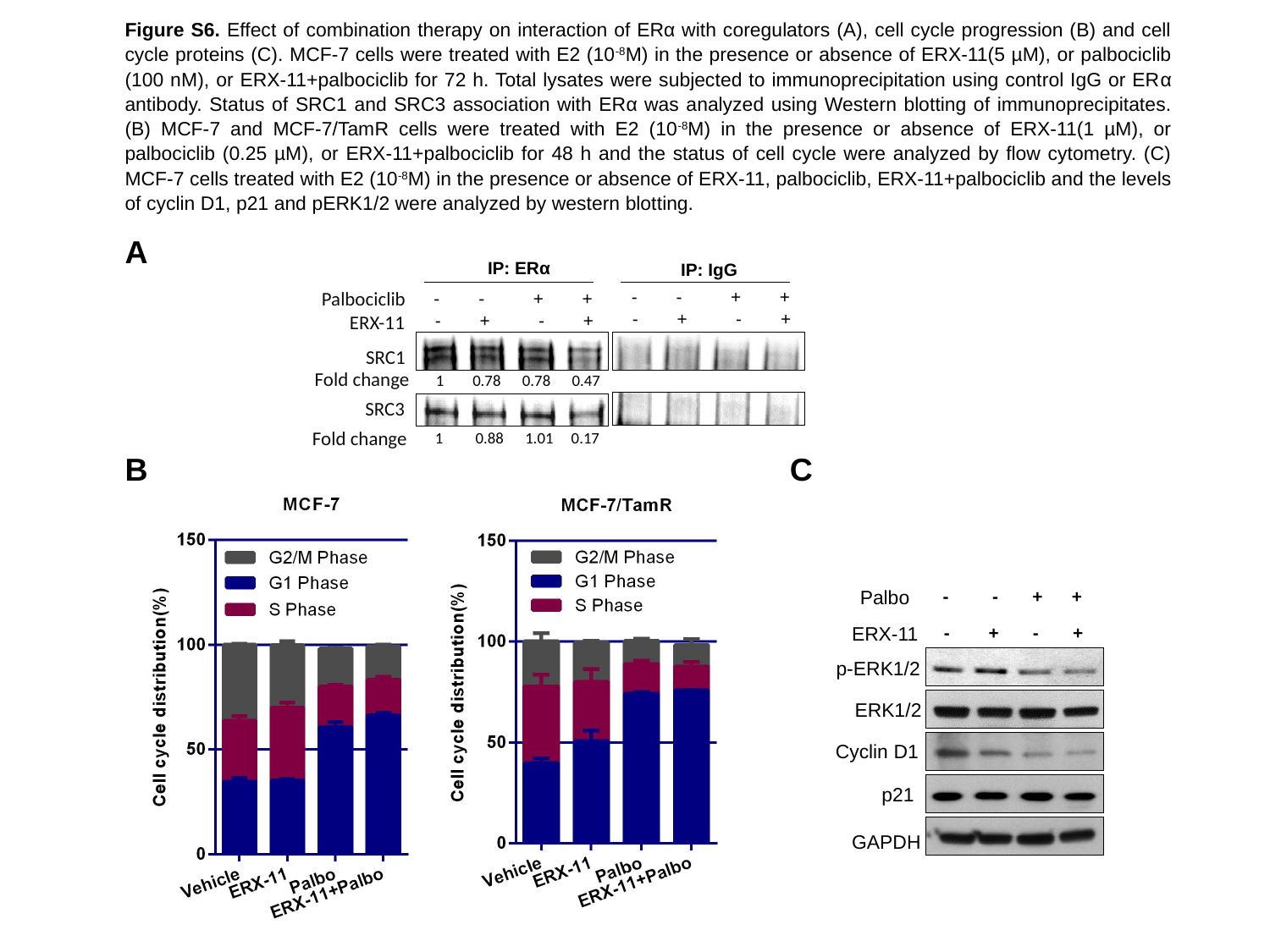

Figure S6. Effect of combination therapy on interaction of ERα with coregulators (A), cell cycle progression (B) and cell cycle proteins (C). MCF-7 cells were treated with E2 (10-8M) in the presence or absence of ERX-11(5 µM), or palbociclib (100 nM), or ERX-11+palbociclib for 72 h. Total lysates were subjected to immunoprecipitation using control IgG or ERα antibody. Status of SRC1 and SRC3 association with ERα was analyzed using Western blotting of immunoprecipitates. (B) MCF-7 and MCF-7/TamR cells were treated with E2 (10-8M) in the presence or absence of ERX-11(1 µM), or palbociclib (0.25 µM), or ERX-11+palbociclib for 48 h and the status of cell cycle were analyzed by flow cytometry. (C) MCF-7 cells treated with E2 (10-8M) in the presence or absence of ERX-11, palbociclib, ERX-11+palbociclib and the levels of cyclin D1, p21 and pERK1/2 were analyzed by western blotting.
A
IP: ERα
- - + +
- + - +
IP: IgG
- - + +
- + - +
Palbociclib
ERX-11
SRC1
Fold change
1 0.78 0.78 0.47
SRC3
Fold change
1 0.88 1.01 0.17
B
C
Palbo
 - - + +
 - + - +
ERX-11
p-ERK1/2
ERK1/2
Cyclin D1
p21
GAPDH

## Slide 7
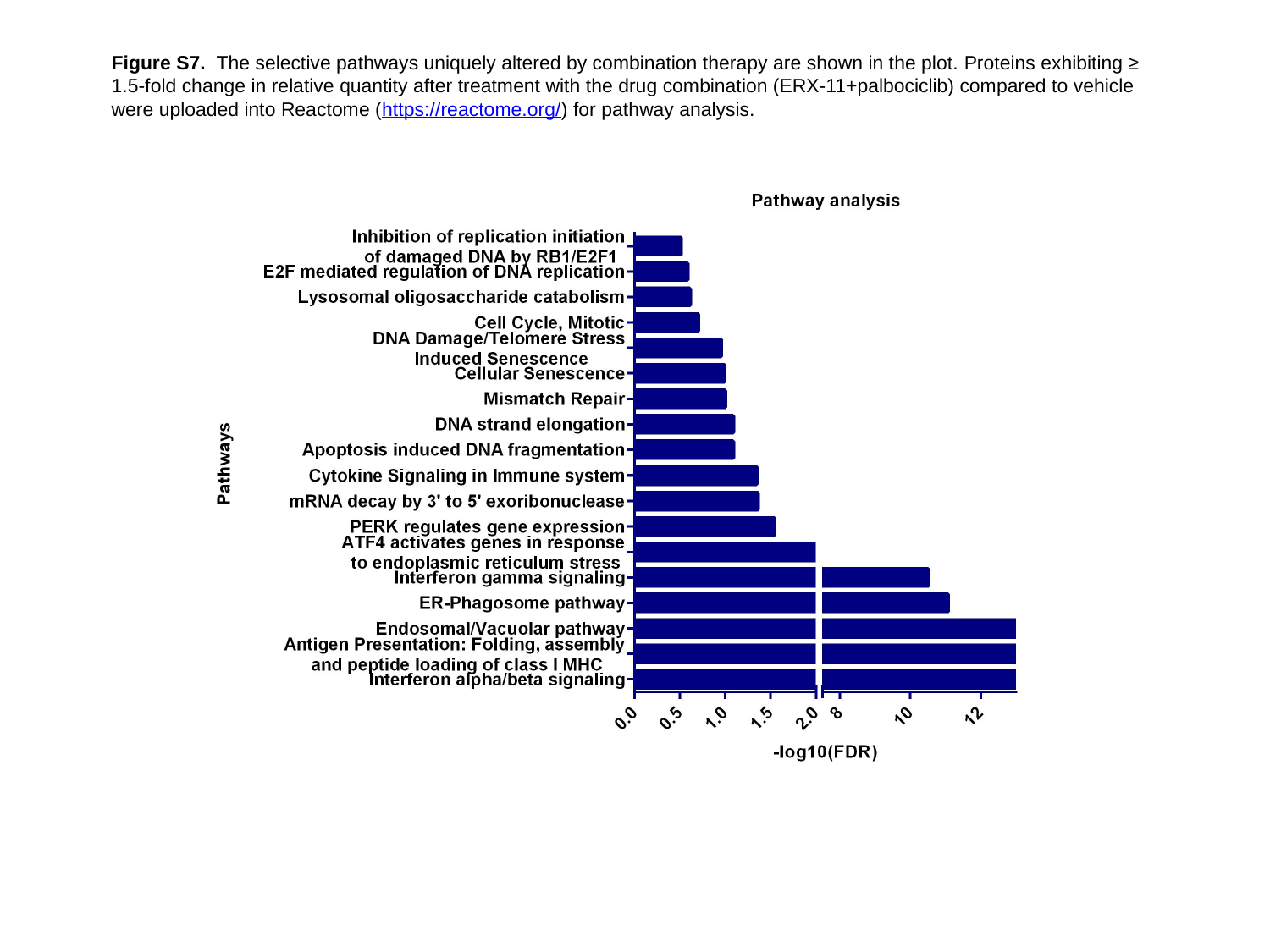

Figure S7. The selective pathways uniquely altered by combination therapy are shown in the plot. Proteins exhibiting ≥ 1.5-fold change in relative quantity after treatment with the drug combination (ERX-11+palbociclib) compared to vehicle were uploaded into Reactome (https://reactome.org/) for pathway analysis.

## Slide 8
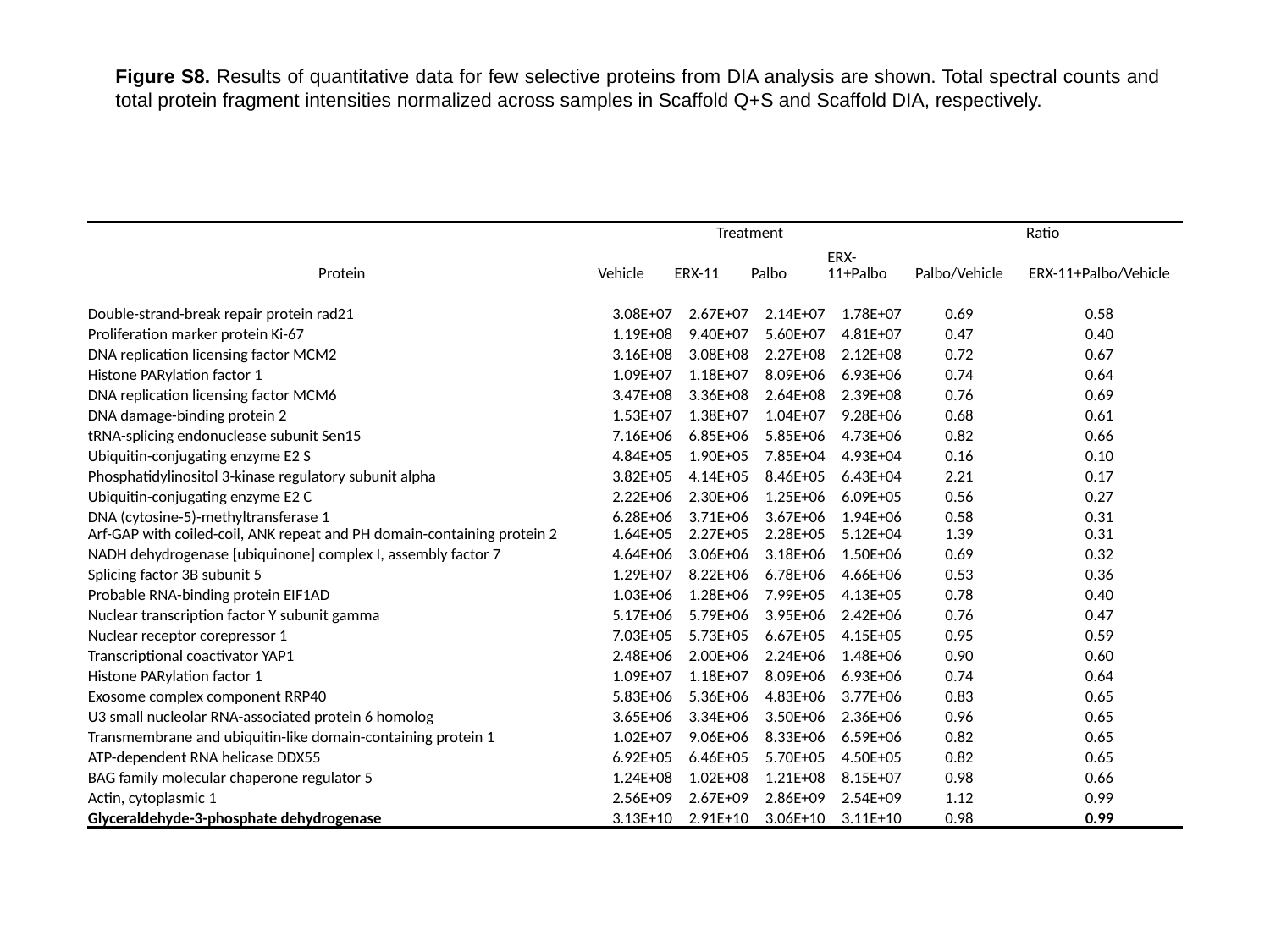

Figure S8. Results of quantitative data for few selective proteins from DIA analysis are shown. Total spectral counts and total protein fragment intensities normalized across samples in Scaffold Q+S and Scaffold DIA, respectively.
| | Treatment | | | | Ratio | |
| --- | --- | --- | --- | --- | --- | --- |
| Protein | Vehicle | ERX-11 | Palbo | ERX-11+Palbo | Palbo/Vehicle | ERX-11+Palbo/Vehicle |
| | | | | | | |
| Double-strand-break repair protein rad21 | 3.08E+07 | 2.67E+07 | 2.14E+07 | 1.78E+07 | 0.69 | 0.58 |
| Proliferation marker protein Ki-67 | 1.19E+08 | 9.40E+07 | 5.60E+07 | 4.81E+07 | 0.47 | 0.40 |
| DNA replication licensing factor MCM2 | 3.16E+08 | 3.08E+08 | 2.27E+08 | 2.12E+08 | 0.72 | 0.67 |
| Histone PARylation factor 1 | 1.09E+07 | 1.18E+07 | 8.09E+06 | 6.93E+06 | 0.74 | 0.64 |
| DNA replication licensing factor MCM6 | 3.47E+08 | 3.36E+08 | 2.64E+08 | 2.39E+08 | 0.76 | 0.69 |
| DNA damage-binding protein 2 | 1.53E+07 | 1.38E+07 | 1.04E+07 | 9.28E+06 | 0.68 | 0.61 |
| tRNA-splicing endonuclease subunit Sen15 | 7.16E+06 | 6.85E+06 | 5.85E+06 | 4.73E+06 | 0.82 | 0.66 |
| Ubiquitin-conjugating enzyme E2 S | 4.84E+05 | 1.90E+05 | 7.85E+04 | 4.93E+04 | 0.16 | 0.10 |
| Phosphatidylinositol 3-kinase regulatory subunit alpha | 3.82E+05 | 4.14E+05 | 8.46E+05 | 6.43E+04 | 2.21 | 0.17 |
| Ubiquitin-conjugating enzyme E2 C | 2.22E+06 | 2.30E+06 | 1.25E+06 | 6.09E+05 | 0.56 | 0.27 |
| DNA (cytosine-5)-methyltransferase 1 | 6.28E+06 | 3.71E+06 | 3.67E+06 | 1.94E+06 | 0.58 | 0.31 |
| Arf-GAP with coiled-coil, ANK repeat and PH domain-containing protein 2 | 1.64E+05 | 2.27E+05 | 2.28E+05 | 5.12E+04 | 1.39 | 0.31 |
| NADH dehydrogenase [ubiquinone] complex I, assembly factor 7 | 4.64E+06 | 3.06E+06 | 3.18E+06 | 1.50E+06 | 0.69 | 0.32 |
| Splicing factor 3B subunit 5 | 1.29E+07 | 8.22E+06 | 6.78E+06 | 4.66E+06 | 0.53 | 0.36 |
| Probable RNA-binding protein EIF1AD | 1.03E+06 | 1.28E+06 | 7.99E+05 | 4.13E+05 | 0.78 | 0.40 |
| Nuclear transcription factor Y subunit gamma | 5.17E+06 | 5.79E+06 | 3.95E+06 | 2.42E+06 | 0.76 | 0.47 |
| Nuclear receptor corepressor 1 | 7.03E+05 | 5.73E+05 | 6.67E+05 | 4.15E+05 | 0.95 | 0.59 |
| Transcriptional coactivator YAP1 | 2.48E+06 | 2.00E+06 | 2.24E+06 | 1.48E+06 | 0.90 | 0.60 |
| Histone PARylation factor 1 | 1.09E+07 | 1.18E+07 | 8.09E+06 | 6.93E+06 | 0.74 | 0.64 |
| Exosome complex component RRP40 | 5.83E+06 | 5.36E+06 | 4.83E+06 | 3.77E+06 | 0.83 | 0.65 |
| U3 small nucleolar RNA-associated protein 6 homolog | 3.65E+06 | 3.34E+06 | 3.50E+06 | 2.36E+06 | 0.96 | 0.65 |
| Transmembrane and ubiquitin-like domain-containing protein 1 | 1.02E+07 | 9.06E+06 | 8.33E+06 | 6.59E+06 | 0.82 | 0.65 |
| ATP-dependent RNA helicase DDX55 | 6.92E+05 | 6.46E+05 | 5.70E+05 | 4.50E+05 | 0.82 | 0.65 |
| BAG family molecular chaperone regulator 5 | 1.24E+08 | 1.02E+08 | 1.21E+08 | 8.15E+07 | 0.98 | 0.66 |
| Actin, cytoplasmic 1 | 2.56E+09 | 2.67E+09 | 2.86E+09 | 2.54E+09 | 1.12 | 0.99 |
| Glyceraldehyde-3-phosphate dehydrogenase | 3.13E+10 | 2.91E+10 | 3.06E+10 | 3.11E+10 | 0.98 | 0.99 |

## Slide 9
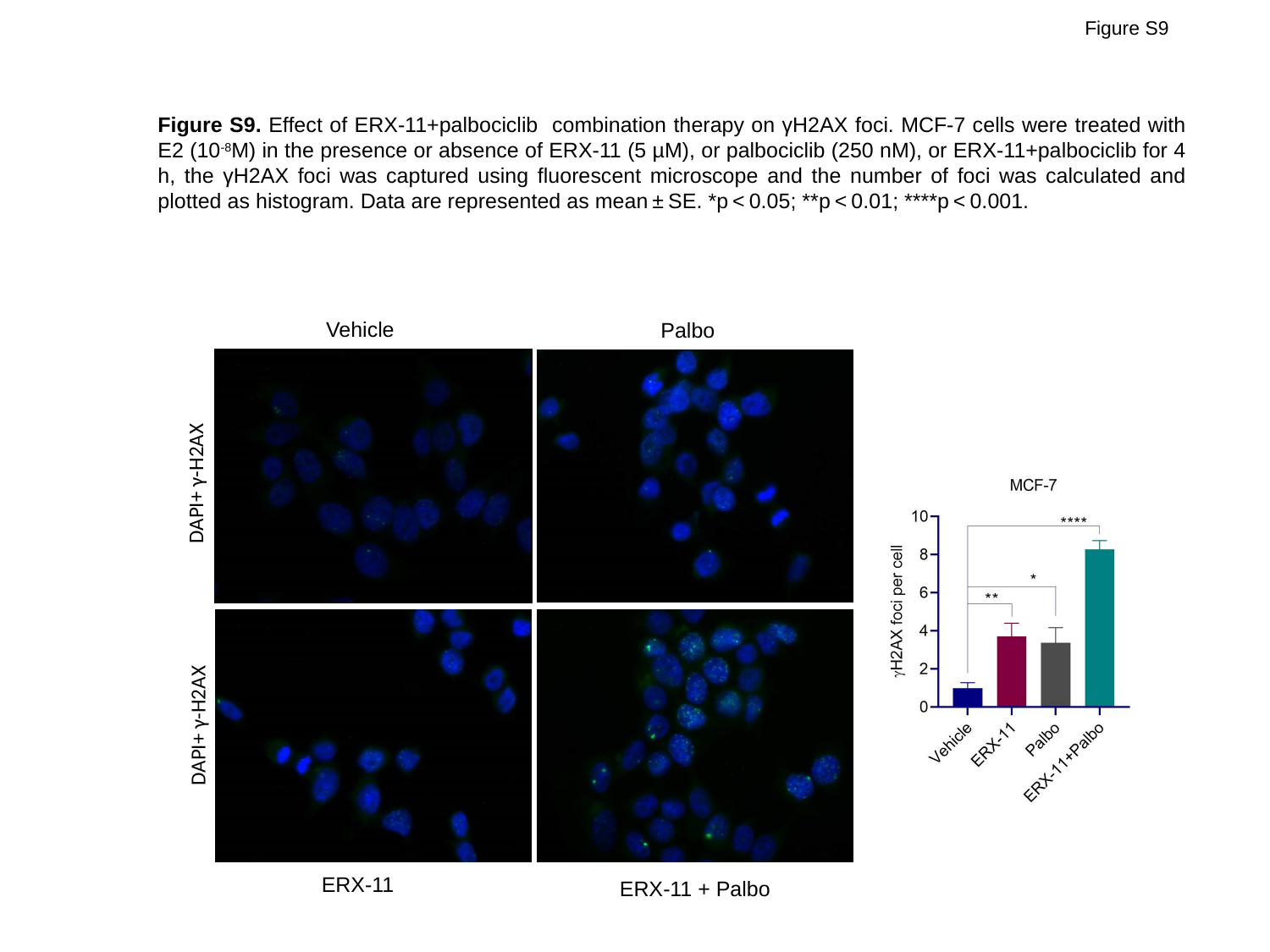

Figure S9
Figure S9. Effect of ERX-11+palbociclib combination therapy on γH2AX foci. MCF-7 cells were treated with E2 (10-8M) in the presence or absence of ERX-11 (5 µM), or palbociclib (250 nM), or ERX-11+palbociclib for 4 h, the γH2AX foci was captured using fluorescent microscope and the number of foci was calculated and plotted as histogram. Data are represented as mean ± SE. *p < 0.05; **p < 0.01; ****p < 0.001.
Vehicle
Palbo
DAPI+ γ-H2AX
ERX-11
ERX-11 + Palbo
DAPI+ γ-H2AX

## Slide 10
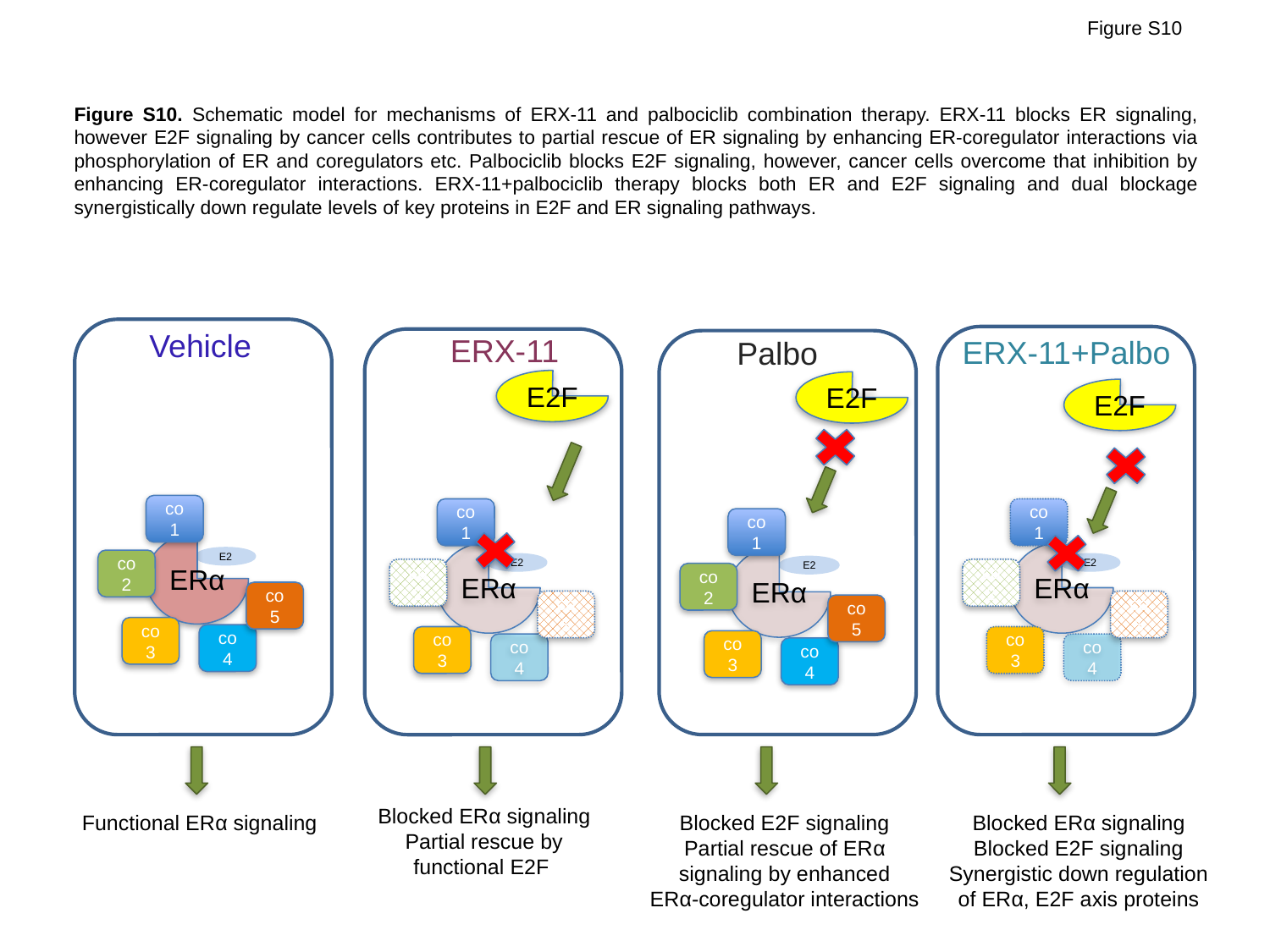

Figure S10
Figure S10. Schematic model for mechanisms of ERX-11 and palbociclib combination therapy. ERX-11 blocks ER signaling, however E2F signaling by cancer cells contributes to partial rescue of ER signaling by enhancing ER-coregulator interactions via phosphorylation of ER and coregulators etc. Palbociclib blocks E2F signaling, however, cancer cells overcome that inhibition by enhancing ER-coregulator interactions. ERX-11+palbociclib therapy blocks both ER and E2F signaling and dual blockage synergistically down regulate levels of key proteins in E2F and ER signaling pathways.
Vehicle
co1
ERα
E2
co2
co5
co3
co4
ERX-11
co1
ERα
E2
co2
co5
co3
co4
ERX-11+Palbo
co1
ERα
E2
co2
co5
co3
co4
Palbo
co1
ERα
E2
co2
co5
co3
co4
E2F
E2F
E2F
Blocked ERα signaling
Partial rescue by
functional E2F
Functional ERα signaling
Blocked E2F signaling
Partial rescue of ERα
signaling by enhanced
ERα-coregulator interactions
Blocked ERα signaling
Blocked E2F signaling
Synergistic down regulation
of ERα, E2F axis proteins
